# Survey of student perception of medical education environment among emergency medicine residents of an academic medical centre in Northern India

**DOI:** 10.1186/s12245-016-0098-3

**Published:** 2016-02-19

**Authors:** Sushant Chhabra, Asit Misra, Sumiyah Shah, Tamorish Kole

**Affiliations:** Department of Emergency Medicine, Max Healthcare, 2 Press Enclave Road, Saket, New Delhi 110017 India; Department of Emergency Medicine, Max Healthcare, Greater Noida, Noida, Uttar Pradesh India; Department of Emergency Medicine, Max Healthcare, Shalimar Bagh, New Delhi India

**Keywords:** Post-graduate training, Emergency medicine, DREEM, Education environment

## Abstract

**Background:**

The specialty of emergency medicine is in its infancy state in the long history of the Indian health sciences education system. Little analytical published data is available at the moment in India regarding the quality of medical education as perceived by the students.

Roff et al. (Med Teach 19: 295–299, 1997) developed a methodology using a Delphi panel to standardize the measurement of medical education known as the Dundee Ready Education Environment Measure (DREEM), which is widely utilized.

The purpose of this survey is to investigate student perceptions of medical education environment among emergency medicine residents of an academic medical centre in Northern India using the DREEM tool.

**Methods:**

The DREEM questionnaire was administered to the students undergoing 3-year post-graduate emergency medicine training in our residency programme. A total of 35 students enrolled from all 3 years of the residency programme completed the survey in May 2013. The results were analysed using STATA 9.0.

**Results:**

The reliability coefficient which was calculated using Cronbach’s alpha for the totality of items of this study was 0.92, which indicates high internal consistency.

The mean (95 % CI) for the overall DREEM was 139.8 (133.3, 146.2), which showed excellent educational environment among the medical students.

**Conclusions:**

The DREEM score is a universal tool for assessment of education provided by health science institutes. With a total score of 139.8, the study conducted at our institute showed comparable results to the original DREEM study conducted by Roff et al. The good scores in all the five subscales reveal an excellent educational programme and learning environment as perceived by the students enrolled at our institution.

## Background

The specialty of emergency medicine is in its infancy state in the long history of the Indian health sciences education system. Only meagre analytical published data is available in India regarding the quality of medical education. In the era of student-oriented educational programmes, it is important to regularly assess the current educational system and update it to the global standards.

Since 1961, medical profession educators [1] have attempted to determine the quality of medical education provided across the globe. With rapidly changing trends in medical education, it has become paramount to measure, identify, quantify and compare the standard of educational institutes from a student’s point of view.

In view of the research conducted by Genn and Harden [2], Roff et al. [3] developed a methodology using a Delphi panel to standardize the measurement of medical education in 1997 [3]. This widely utilized methodology is the Dundee Ready Education Environment Measure (DREEM). The DREEM is divided into five subscales relating students’ perception of learning, perception of teachers, academic self-perception, perception of atmosphere and student’s social self-perception. The students are scored for 50 items with a global score of 200. Since its initial publication, DREEM has been validated by multiple studies to be a reliable and consistent methodology for assessment of various aspects of medical education across all the subsets of the healthcare profession institutes internationally.

Brown et al. [4] conducted a large-scale DREEM study in Australia in 2011 consisting of 548 participants from most major health science courses in Monash University, Victoria. Their study showed a positive perception among the students towards the medical education and learning environment with a fairly high score (mean, 137.3; standard deviation (SD), 18.3). It also gave a good comparison of the similarities and differences among the various health science disciplines.

Another study conducted in 2010 at the University of Colombo, faculty of medicine in Sri Lanka, by Lokuhetty et al. [5] on senior undergraduates after introduction of a new curriculum showed that though the score was comparable to the previous study in the country it was a lower score than most studies (mean, 107) revealing numerous deficiencies in their educational methodology.

In a study conducted among chiropractic students in University of Dundee by Palmgren and Chandratilake [6] reported the highest published score yet (mean, 156.7; SD, 3.7). The study revealed the excellent level of student-perceived quality of the chiropractic training institute despite demographic variations.

The validity of the DREEM score was tested among Malaysian medical students by Yusoff [7]. The five-factor structure was found to be unsupportive; however, a shortened version gave scores comparable to the original score.

A psychometric appraisal of the DREEM score by Hammond et al. [8] questioned the internal consistency of the five-scale model and the construct validity. It suggested a revision of the model for current educational methodology and integration of recent multinational analyses to provide comparability among institutes across the globe.

With the application of the DREEM to an institution in India, the main aim would be to evaluate whether the current educational system provides a student satisfactory learning environment and to analyse the variables responsible for the discrepancies if present. This would also lay down a path for future researchers and create a database of reference in the Indian context.

### Objectives

The purpose of this survey is to investigate student perceptions of medical education environment among emergency medicine residents of an academic medical centre in Northern India using DREEM tool.

## Methods

A survey was conducted among the students undergoing post-graduate emergency medicine training in tertiary care academic medical centre in Northern India. It is a 3-year post-graduate programme in emergency medicine in collaboration with a US university. A total of 35 candidates (out of 38) completed the survey. The survey was conducted anonymously with every batch being given the questionnaire at the end of a regular class after a brief introduction of the study. Consent from all the students was taken before giving them the questionnaire.

### Study instrument

DREEM is a questionnaire with 50 items that assess five domains [3]: students’ perceptions of learning, 12 items, and maximum score 48; students’ perceptions of teachers, 11 items, maximum score 44; students’ academic self-perception, 8 items, maximum score 32; students’ perceptions of atmosphere, 12 items, maximum score 48; and students’ social self-perception, 7 items, maximum score 28. Each item is rated on a five-point Likert scale from 0 to 4 where 0 = strongly disagree, 1 = disagree, 2 = unsure, 3 = agree and 4 = strongly agree. There are nine negative items (items 4, 8, 9, 17, 25, 35, 39, 48 and 50), for which correction is made by reversing the scores; thus, after correction, higher scores indicate disagreement with that item. Items with a mean score of ≥3 are true positive points; those with a mean of ≤2 are problem areas; scores in between these two (between 2 and 3) limits indicate aspects of the environment that could be enhanced. The maximal global score for the questionnaire is 200, and the global score is interpreted as follows: 0–50 = very poor, 51–100 = many problems; 101–150 = more positive than negative and 151–200 = excellent.

The data was collected and entered into a Microsoft Excel spreadsheet (Microsoft Co., Redmond, WA, USA); Stata 9.0 (College Station, Texas, USA) was used for analysis of the data. Calculation of mean and standard deviation was done.

## Results

The reliability coefficient was calculated using Cronbach’s alpha. Cronbach’s alpha for the totality of items was 0.92, which indicates high internal consistency. Cronbach’s alpha for students’ perception of learning, students’ perception of teachers, students’ academic self-perception, students’ perception of atmosphere and students’ social self-perception were 0.79, 0.77, 0.71, 0.81 and 0.52, respectively.

**Table:** 

*Domains*	*Number of items*	*Maximum score*	*Mean ± SD*	*95 % CI*	*Cronbach’s alpha*
Students’ perception of learning	12	48	33.6 ± 5.1	(31.9, 35.4)	0.79
Students’ perception of teachers	11	44	29.7 ± 4.9	(27.9, 31.4)	0.77
Students’ academic self-perception	8	32	23.6 ± 3.3	(22.5, 24.8)	0.71
Students’ perception of atmosphere	12	48	34.0 ± 5.6	(32.1, 35.9)	0.81
Students’ social self-perception	7	28	18.8 ± 3.5	(17.6, 20.0)	0.52
Overall	50	200	139.8 ± 18.8	(133.3, 146.2)	0.92

The mean (95 % CI) for the overall DREEM was 139.8 (133.3, 146.2), which showed excellent educational environment among the medical students.

The following table describes the average scores in different domains:

**Table:** 

Question no.	Mean + SD	Strength/weakness
*Students’ perception of learning*		
Q1	3.3 ± 0.58	Real positive point
Q7	2.9 ± 0.57	Needs enhancement
Q13	2.5 ± 0.88	Needs enhancement
Q16	3.5 ± 0.70	Real positive point
Q20	3.0 ± 0.66	Real positive point
Q21	3.2 ± 0 .76	Real positive point
Q24	2.8 ± 0.78	Needs enhancement
Q25	1.6 ± 0.85	Problem areas
Q38	3.0 ± 0.68	Real positive point
Q44	2.9 ± 0.64	Needs enhancement
Q47	2.5 ± 0.95	Needs enhancement
Q48	2.3 ± 0.97	Needs enhancement
*Students’ perception of teachers*		
Q2	3.4 ± 0.60	Real positive point
Q6	2.8 ± 0.66	Needs enhancement
Q8	2. 2 ± 0.95	Needs enhancement
Q9	1.8 ± 1.0	Problem areas
Q18	2.9 ± 0.68	Needs enhancement
Q29	2.6 ± 0.85	Needs enhancement
Q32	2.6 ± 0.91	Needs enhancement
Q37	2.9 ± 0.70	Needs enhancement
Q39	2.6 ± 0.94	Needs enhancement
Q40	3.0 ± 0.51	Real positive point
Q49	2.8 ± 0.99	Needs enhancement
*Students’ academic self-perception*		
Q5	2.6 ± 0.77	Needs enhancement
Q10	3.0 ± 0.76	Real positive point
Q22	3.3 ± 0.59	Real positive point
Q26	2.9 ± 0.66	Needs enhancement
Q27	2.4 ± 0.95	Needs enhancement
Q31	3.0 ± 0.68	Real positive point
Q41	3.1 ± 0.61	Real positive point
Q45	3.1 ± 0.72	Real positive point
*Students’ perception of atmosphere*		
Q11	2.9 ± 0.78	Needs enhancement
Q12	2.7 ± 0.78	Needs enhancement
Q17	3.0 ± 0.92	Real positive point
Q23	3.1 ± 0.53	Real positive point
Q30	3.1 ± 0.73	Real positive point
Q33	3.1 ± 0.51	Real positive point
Q34	3.0 ± 0.54	Real positive point
Q35	3.0 ± 0.97	Real positive point
Q36	2.7 ± 1.02	Needs enhancement
Q42	1.9 ± 0.98	Problem areas
Q43	2.7 ± 0.86	Needs enhancement
Q50	2.5 ± 0.98	Needs enhancement
*Students’ social self-perception*		
Q3	2.1 ± 1.06	Needs enhancement
Q4	1.8 ± 1.22	Problem areas
Q14	2.5 ± 1.07	Needs enhancement
Q15	3. 5 ± 0.61	Real positive point
Q19	3.1 ± 0.91	Real positive point
Q28	2.7 ± 1.12	Needs enhancement
Q46	2.9 ± 0.89	Needs enhancement

Among the 50 items, 4 items were found to be problematic areas with scores of <2. Issues that required immediate attention were the students were too tired to enjoy the course, the teachers were being quite authoritarian and an overemphasis of factual learning during the programme with the stress of work being overwhelming. Multiple other areas with scores between 2 and 3 identified that the creation of a more student-friendly environment be given consideration for planning the curriculum of future generations.

## Discussion

The internationally validated DREEM score is a universal tool for assessment of education provided by health science institutes. It transgresses the cultural boundaries and despite being subject to multiple tests has withstood its basic functionality. However, it needs to be analysed in depth regarding the possibility of a shortened version by elimination of few questionable links in the original score.

With a total score of 139.8, the study conducted at our institute showed comparable results to the original DREEM study conducted by Roff et al. [3]. The good scores in all the five subscales reveal an excellent educational programme and learning environment as perceived by the students enrolled at this institution.

The study has drawn attention to a few aspects of the institute that need to be revised in order to provide a better student-centred educational atmosphere. The problematic aspects can be tackled by introducing a curriculum that included problem-based learning, structured bedside clinical teaching with the specific objective of mentoring students by faculties on a day-to-day basis, (like daily discussions, morning meetings), so that students feel more free in expressing their problems and things are tackled in a better way.

## Conclusions

This study identified the following problematic areas with this group of students:

1. Teaching overemphasized factual learning.
2. Students’ perception of the course organizers being authoritarian.
3. Perception was that students were too tired to enjoy the course.
  Earlier, two studies have been conducted in India in two medical council of India-recognized government medical colleges to evaluate their educational environment using DREEM, both of which showed that considerable improvement is required across all domains of educational environment, while ours was a study which was first of a kind as it was done in a private academic medical institution which runs an emergency medicine post-graduate programme in collaboration with a US university. The results of our study were quite encouraging with mean total score of 139.8 which establishes the fact that an educational environment such as this can be created to fulfil the enormous requirement of emergency physicians to serve the 1.2 billion people of India.

- There is a need to conduct similar studies in different institutions affiliated to one academic programme or different programmes under the speciality of emergency medicine.

## Abbreviations

CI: confidence interval
DREEM: Dundee Ready Education Environment Measure
SD: standard deviation
